# Peer Review in a General Medical Research Journal Before and During the COVID-19 Pandemic

**DOI:** 10.1001/jamanetworkopen.2022.53296

**Published:** 2023-01-03

**Authors:** Roy H. Perlis, Jacob Kendall-Taylor, Kamber Hart, Ishani Ganguli, Jesse A. Berlin, Steven M. Bradley, Sebastien Haneuse, Sharon K. Inouye, Elizabeth A. Jacobs, Arden Morris, Olugbenga Ogedegbe, Eli Perencevich, Lawrence N. Shulman, N. Seth Trueger, Stephan D. Fihn, Frederick P. Rivara, Annette Flanagin

**Affiliations:** Massachusetts General Hospital, Harvard Medical School, Boston; JAMA Network, Chicago, Illinois; Harvard Medical School, Boston, Massachusetts; Brigham and Women’s Hospital, Harvard Medical School, Boston, Massachusetts; Rutgers School of Public Health, Piscataway, New Jersey; Minneapolis Heart Institute, Minneapolis Heart Institute Foundation, Minneapolis, Minnesota; Harvard T.H. Chan School of Public Health, Boston, Massachusetts; Hebrew SeniorLife and Beth Israel Deaconess Medical Center, Harvard Medical School, Boston, Massachusetts; MaineHealth and Maine Medical Center Research Institute, Scarborough; Stanford University School of Medicine, Stanford, California; NYU Langone Health, New York, New York; Carver College of Medicine, University of Iowa, Iowa City; Abramson Cancer Center, University of Pennsylvania, Philadelphia; Department of Emergency Medicine, Northwestern University Feinberg School of Medicine, Chicago, Illinois; *JAMA Network Open*, Chicago, Illinois; University of Washington School of Medicine, Seattle; University of Washington School of Medicine, Seattle; JAMA Network, Chicago, Illinois

## Abstract

**IMPORTANCE:**

Although peer review is an important component of publication for new research, the viability of this process has been questioned, particularly with the added stressors of the COVID-19 pandemic.

**OBJECTIVE:**

To characterize rates of peer reviewer acceptance of invitations to review manuscripts, reviewer turnaround times, and editor-assessed quality of reviews before and after the start of the COVID-19 pandemic at a large, open-access general medical journal.

**DESIGN, SETTING, AND PARTICIPANTS:**

This retrospective, pre-post cohort study examined all research manuscripts submitted to *JAMA Network Open* between January 1, 2019, and June 29, 2021, either directly or via transfer from other JAMA Network journals, for which at least 1 peer review of manuscript content was solicited. Measures were compared between the period before the World Health Organization declaration of a COVID-19 pandemic on March 11, 2020 (14.3 months), and the period during the pandemic (15.6 months) among all reviewed manuscripts and between pandemic-period manuscripts that did or did not address COVID-19.

**MAIN OUTCOMES AND MEASURES:**

For each reviewed manuscript, the number of invitations sent to reviewers, proportions of reviewers accepting invitations, time in days to return reviews, and editor-assessed quality ratings of reviews were determined.

**RESULTS:**

In total, the journal sought review for 5013 manuscripts, including 4295 Original Investigations (85.7%) and 718 Research Letters (14.3%); 1860 manuscripts were submitted during the prepandemic period and 3153 during the pandemic period. Comparing the prepandemic with the pandemic period, the mean (SD) number of reviews rated as high quality (very good or excellent) per manuscript increased slightly from 1.3 (0.7) to 1.5 (0.7) (*P* < .001), and the mean (SD) time for reviewers to return reviews was modestly shorter (from 15.8 [7.6] days to 14.4 [7.0] days; *P* < .001), a difference that persisted in linear regression models accounting for manuscript type, study design, and whether the manuscript addressed COVID-19.

**CONCLUSIONS AND RELEVANCE:**

In this cohort study, the speed and editor-reported quality of peer reviews in an open-access general medical journal improved modestly during the initial year of the pandemic. Additional study will be necessary to understand how the pandemic has affected reviewer burden and fatigue.

## Introduction

The effect of COVID-19 on academic publishing has been the subject of substantial discussion. In particular, the pandemic has reinvigorated conversations about the growing role and variable quality of preprints that do not undergo peer review,^1^ the burden of peer review on the academic community,^2^ concerns about reviewer fatigue,^3^ and how best to ensure the rigor and value of peer-reviewed medical literature.^4,5^ For medical publishing specifically, increasing volumes of manuscripts related to COVID-19^6–8^ and expectations for rapid publication and dissemination have further stressed a system that some in medicine already believed was broken.^9^

Few empirical observations about the ways in which the peer review process may have changed during the pandemic have been reported. A recent study^7^ of manuscripts and reviews submitted to 2329 journals before and during the pandemic found a modest decrease in rates of reviewer acceptance of invitations to review among health and medical journals between February and May 2020, with a more pronounced decrease among potential reviewers who were women, but not men.

To understand how peer review changed with the onset of the COVID-19 pandemic, we examined peer review data from *JAMA Network Open*, an open-access general medical journal launched in 2018 with a 2020 impact factor of 8.5.^10^ Specifically, we aimed to quantify changes in rates of peer reviewer acceptance of invitations to review manuscripts and review quality from the period before to the period during the COVID-19 pandemic.

## Methods

### Manuscript Review Process and Data Set Generation

Manuscripts submitted to *JAMA Network Open* first undergo technical quality assessment, then close evaluation by an editor. For manuscripts that are deemed of sufficient quality and priority to undergo peer review, editors seek review from 1 or more content reviewers and 1 statistical reviewer. We extracted data from databases used by the JAMA Network to track manuscript submissions and peer reviews. We included all manuscripts received at *JAMA Network Open* from January 1, 2019, through June 30, 2021, that were categorized as Original Investigations or Research Letters for which at least 1 content review was sought. These manuscripts could be submitted directly to the journal or transferred from other journals within the JAMA Network. The study used deidentified administrative data with no participant contact and followed the journal’s policy for such research, which indicates that information may be systematically collected and analyzed as part of research to improve the quality of the editorial or peer review process. This study was reviewed by the Massachusetts General–Brigham Institutional Review Board and considered to be exempt from informed consent because it uses deidentified data and posed minimal risk. This study followed the Strengthening the Reporting of Observational Studies in Epidemiology (STROBE) reporting guideline for cohort studies.^11^

### Measures

For each manuscript, we determined the total number of individuals who were invited to review the manuscript and the number and proportion of reviewers accepting, declining, or failing to respond to invitations. For the accepted invitations, we determined the mean time (across reviewers) to return the review and the number and proportion of reviews rated by editors as very good or excellent on a 5-point anchored scale: poor, fair, good, very good, or excellent. This process was completed for all manuscripts as part of routine editorial practice. For purposes of analysis, the last 2 categories (very good and excellent) were aggregated to reflect high-quality reviews. For descriptive purposes and because some individuals served as peer reviewers for multiple manuscripts, we also report acceptance rate for reviewer invitations, time to return review, and quality of review at the level of individual reviews.

Additional manuscript features collected at submission included study design^12^ (eg, randomized trial, cohort study, or economic evaluation), whether a study was submitted directly or transferred from another JAMA Network journal, and whether a study referenced or addressed COVID-19. The last of these is determined via an automated process implemented to identify any manuscript with any of the following terms in the manuscript text: *acute respiratory virus, personal protective equipment, N95, COVID, COVID-19, coronavirus, SARS, or novel virus.*

### Statistical Analysis

We compared peer review characteristics for submitted manuscripts with an index date before or after March 11, 2020, the date that the World Health Organization declared a COVID-19 pandemic.^13^ Thus, data were divided into 2 groups: the prepandemic period from January 1, 2019, to March 10, 2020 (14.3 months), and the pandemic period from March 11, 2020, to June 29, 2021 (15.6 months). We similarly compared characteristics of reviews provided for manuscripts submitted after March 11, 2020, that did or did not address COVID-19. We used multivariable linear regression to calculate effect sizes and 95% CIs for the association between prepandemic and pandemic status for reviewer turnaround time, adjusted for other manuscript characteristics (study design, direct submission vs transfer from other JAMA Network journals, article type, and whether the manuscript addressed COVID-19). (Incorporating clustering by subject area, as a proxy for a handling editor, did not meaningfully change results.)

To visualize changes in peer review characteristics over time—and, in particular, whether secular trends in these characteristics might have preceded the pandemic—we also described manuscript and review features on a weekly basis, with each manuscript assigned to the date of first reviewer invitation. For graphic presentation, we applied a 3-week rolling mean using the rollmean function in R’s zoo library, version 1.8–9.

All analyses used R software, version 4.1.2^14^; the threshold for statistical significance was considered to be a 2-tailed *P* < .05, without adjustment for multiple comparisons.

## Results

Between January 1, 2019, and June 30, 2021, the journal sought reviews for 5013 manuscripts (mean [SD], 38.3 [13.3] per week), including 4295 Original Investigations (85.7%) and 718 Research Letters (14.3%). Of these manuscripts, 1860 and 3153 manuscripts were submitted during the prepandemic and pandemic periods, respectively.

Characteristics of manuscripts received before March 11, 2020, or on or after that date are summarized in Table 1. These manuscripts included 376 clinical trials (7.5%), 1939 cohort studies (38.7%), and 1148 cross-sectional studies (22.9%); among the 5013 manuscripts reviewed, 932 (18.6%) addressed COVID-19. Table 1 also includes univariate comparisons of these and other characteristics between the prepandemic and pandemic periods. The overall mean (SD) volume of manuscripts reviewed per week increased from 30.3 (8.6) to 46.4 (12.2) (*P* < .001), and the mean (SD) number of reviewers invited per manuscript to achieve the minimum number of required reviews increased from 6.0 (3.6) to 7.0 (4.5) (*P* < .001). The mean (SD) proportion of reviewers per manuscript who accepted invitations did not change significantly (39.5% [28.6%] vs 38.4% [28.3%]; *P* = .21). However, the mean (SD) number of reviews returned per manuscript also increased from 1.6 (0.6) to 1.7 (0.5) (*P* < .001), as did the mean (SD) number of reviews rated as high quality (ie, very good or excellent) per manuscript (from 1.3 [0.7] to 1.5 [0.7]; *P* < .001). Mean (SD) time to return reviews also decreased from 15.8 (7.6) to 14.4 (7.0) days (*P* < .001).

**Figure 1.**
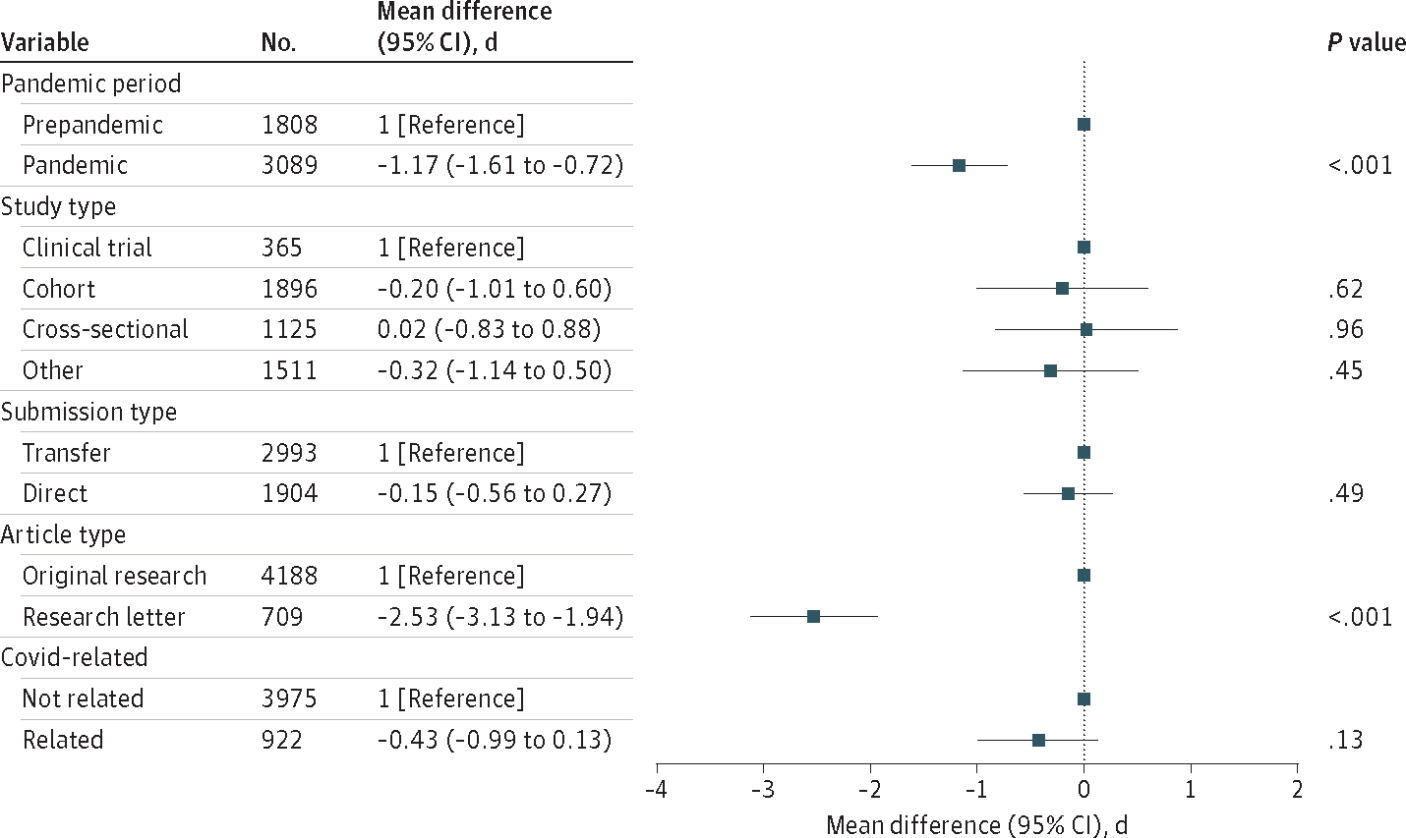
Linear Regression Model of Manuscript Features Associated With Mean Time to Return Reviews Before and During the First Year of the COVID-19 Pandemic Linear regression model of mean time in days to return review and variables associated with mean time in days to return reviews, by manuscript.

In multivariable linear regression adjusting for baseline manuscript features (article type, study design, and direct submission vs transfer), differences in mean time to return review persisted. Time to return of review was decreased in the pandemic period compared with the prepandemic period (adjusted mean difference of −1.2 days; 95% CI, −0.7 to −1.6) (Figure 1).

In a complementary analysis of 33 615 reviewers invited before (n = 13 208 [39.3%]) or after (n = 20 407 [60.7%]) pandemic onset who returned reviews, the proportion of reviewers accepting invitations was similar (3337 [25.3%] vs 5229 [25.6%]; *P* = .46), whereas the mean (SD) time to return reviews decreased from 15.2 (9.2) in the prepandemic period to 14.8 (8.6) during the pandemic (*P* = .02).

A similar pattern of differences to those observed comparing the prepandemic vs pandemic periods was identified in comparisons of manuscripts that did not address COVID-19 (n = 2238) and those that did address COVID-19 (n = 915) (Table 2). COVID-19–related manuscripts required fewer reviewer invitations (mean [SD], 6.4 [4.4] vs 7.2 [4.6]; *P* < .001), and the proportion of reviewers who declined invitations to review was lower (mean [SD], 32.8% [22.6%] vs 35.2% [22.3%]; *P* = .006).

The mean (SD) number of very good or excellent reviews was greater for COVID-19–related manuscripts (1.5 [0.7] vs those not related to COVID-19 (1.4 [0.7]; *P* < .001). Mean (SD) time to return reviews was also lower: 14.6 (7.0) days for those not COVID related vs 13.7 (6.8) days for those that were COVID related (*P* = .002).

**Figure 2.**
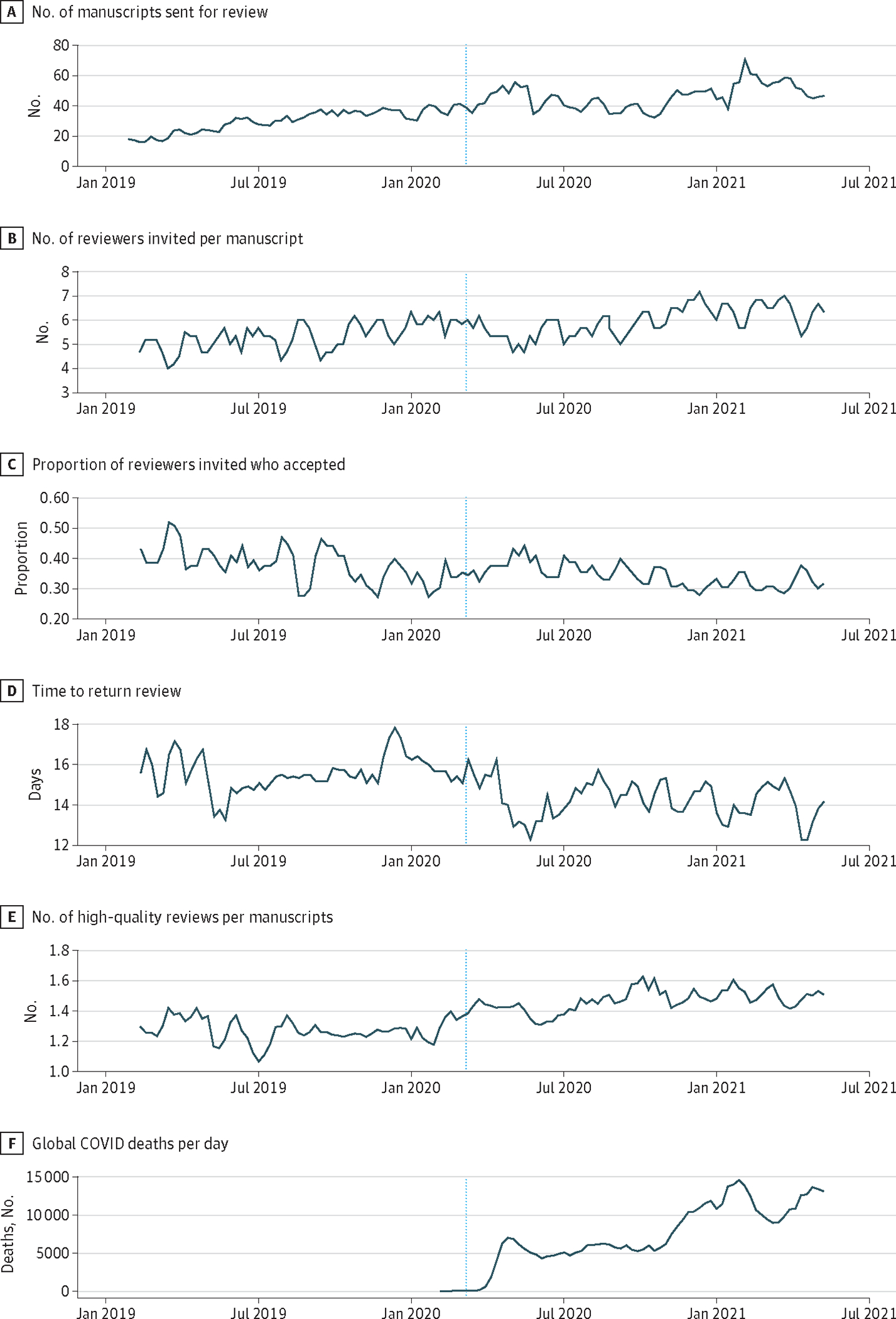
Change in Characteristics of Peer Review Over Time Before and During the First Year of the COVID-19 Pandemic Changes in aspects of peer reviewer behavior over time, before and during the COVID-19 pandemic, with number of global daily deaths from COVID-19 as an indicator of pandemic intensity.

Figure 2 illustrates changes in peer reviews over time, with the global number of COVID-19 deaths at the bottom for reference.^15^ In general, the volume of manuscript submissions increased over time, as did the number of reviewers invited, both continuing qualitative trends beginning before the pandemic. Conversely, a decrease in time to return reviews appears to have followed pandemic onset.

## Discussion

In this cohort study of 5013 manuscripts with reviews solicited by an open-access general medical journal before and during the COVID-19 pandemic, we did not identify evidence of deterioration in the peer review process. The overall rate of reviewer acceptance of review requests remained stable after pandemic onset. However, the time required for reviewers to complete reviews was modestly shorter during the pandemic, and the mean number of high-quality reviews per manuscript was greater compared with the period before the pandemic. Although the association with time to return reviews persisted after adjustment for potential confounding features, such as study design and manuscript type, we cannot exclude unobserved changes in how the editors invited reviewers—for example, making a greater effort to identify interested reviewers or sending personal notes—that may have coincided with the pandemic.

Similar patterns were observed for COVID-19–focused manuscripts. In this case, rates of reviewer acceptance were significantly greater for such manuscripts. Time to return reviews was slightly but statistically significantly shorter, whereas the number of high-quality reviews received per manuscript was slightly greater.

Our results complement those of a recent investigation^7^ of peer review during the pandemic. That study found lower rates of review invitation acceptance from health and medical journals among women but not men.^7^

### Limitations

This study has multiple limitations. First, we did not have access to reviewer-level characteristics, such as gender, age, race and ethnicity, or academic seniority, that would allow us to quantify the differential effect of the pandemic on some reviewers or compare across demographic characteristics, because these measures are not collected by the journal. Second, we cannot necessarily attribute the changes we observed to the pandemic itself. While *JAMA Network Open* was publishing for 2 years before the pandemic, submissions increased rapidly, particularly in the first year, as journal recognition and reputation increased. Likewise, reviewers’ willingness to participate in peer review may have changed over time as they became more aware of the journal. As such, there are likely secular trends that may explain some of the changes we observed; the generalizability of our results to other journals will require further study, and we hope this work will encourage such efforts. Third, editors’ estimates of review quality are entirely subjective, so it is possible that editors took extenuating circumstances into account; for example, editors may have “graded on a curve” during the pandemic, and in reality quality could have remained unchanged or diminished. Finally, the automated flagging of submissions as COVID-19 related may have missed relevant submissions that did not include specific keywords and, likewise, may have flagged submissions that included pandemic-related keywords in the introduction or discussion but were not truly related to COVID-19.

In contextualizing these findings, it is also important to consider other changes in the peer review process occurring during the pandemic. For example, reviewers may have been more or less likely to accept invitations to review manuscripts based on eagerness to review new science about COVID-19, availability due to reduced clinical commitments, unavailability due to intensified clinical commitments, concerns about COVID-related misinformation, or reviewer fatigue associated with increase in volume of manuscripts submitted during the pandemic. Moreover, the editors’ behavior may have changed with the increase in number of submissions, such as making more personal requests or opting to proceed with a decision with fewer reviewers for a given manuscript.

Despite these limitations, our results may help inform ongoing conversations about quality and burden of peer review during the COVID-19 pandemic. The findings suggest that the pandemic modestly affected the review process in terms of turnaround time and review quality. However, this apparent stability does not address the extent to which reviewer sentiment toward the peer review process may have shifted or the pandemic’s effect on the ability of invited reviewers to complete other tasks. Other lines of investigation, including surveys, suggest that the pandemic has negatively affected researchers’ quality of life, more so for women than men.^16,17^

## Conclusions

The findings of this pre-post cohort study suggest that the peer review process at a large, open-access journal has continued to function during the COVID-19 pandemic despite changes in both the volume of submissions and the work and home environments of many peer reviewers. Most encouragingly, during the pandemic, review quality did not appear to have diminished. Still, in light of abundant evidence that COVID-19 has negatively impacted researchers,^16,17^ continued efforts to study and improve the peer review process are needed.

## Supplementary Material

Supplement

## Figures and Tables

**Table 1.** Characteristics of Manuscripts Received Before or on March 11, 2020, or Later

Characteristic	Before pandemic (n = 1860)	During pandemic (n = 3153)	Total (N = 5013)	*P* value^a^
Manuscripts reviewed per week, No.				
Mean (SD)	30.3 (8.6)	46.4 (12.2)	38.3 (13.3)	<.001
Median (IQR)	31 (24–36)	45 (37–53)	37 (28–46)	
Type of submission, No. (%)				
Transfer	1117 (60.1)	1952 (61.9)	3069 (61.2)	.19
Direct submission	743 (39.9)	1201 (38.1)	1944 (38.8)	
Type of article, No. (%)				
Original Investigation	1689 (90.8)	2606 (82.7)	4295 (85.7)	<.001
Research Letter	171 (9.2)	547 (17.3)	718 (14.3)	
Study design, No. (%)				
Clinical trial	150 (8.1)	226 (7.2)	376 (7.5)	.13
Cohort	683 (36.7)	1256 (39.8)	1939 (38.7)	
Cross-sectional	430 (23.1)	718 (22.8)	1148 (22.9)	
Other design	597 (32.1)	953 (30.2)	1550 (30.9)	
Reviewers invited, No.				
Mean (SD)	6.0 (3.6)	7.0 (4.6)	6.6 (4.3)	<.001
Median (IQR)	5.0 (3.0–8.0)	6.0 (4.0–9.0)	6.0 (3.0–9.0)	
Reviewers who accepted, No.				
Mean (SD)	1.6 (0.6)	1.8 (0.5)	1.7 (0.5)	<.001
Median (IQR)	2.0 (1.0–2.0)	2.0 (1.0–2.0)	2.0 (1.0–2.0)	
Invitation acceptance rate, %				
Mean (SD)	39.5 (28.6)	38.4 (28.3)	38.8 (28.4)	.21
Median (IQR)	33.3 (16.7–50.0)	28.6 (16.7–50.0)	28.6 (16.7–50.0)	
Reviewers who declined, No.				
Mean (SD)	2.4 (2.4)	3.0 (2.9)	2.8 (2.7)	<.001
Median (IQR)	2.0 (1.0–4.0)	2.0 (1.0–4.0)	2.0 (1.0–4.0)	
Invitation decline rate, %				
Mean (SD)	33.0 (23.3)	34.5 (22.4)	34.0 (22.8)	.02
Median (IQR)	33.3 (14.3–50.0)	37.5 (20.0–50.0)	33.3 (16.7–50.0)	
Reviews returned, No.				
Mean (SD)	1.6 (0.6)	1.7 (0.5)	1.7 (0.5)	<.001
Median (IQR)	2.0 (1.0–2.0)	2.0 (1.0–2.0)	2.0 (1.0–2.0)	
Mean review turnaround time, d^b^				
Mean (SD)	15.8 (7.6)	14.4 (7.0)	14.9 (7.2)	<.001
Median (IQR)	15.5 (11.0–20.0)	14.0 (10.0–18.0)	15.0 (10.0–19.0)	
Very good or excellent reviews, No.				
Mean (SD)	1.3 (0.7)	1.5 (0.7)	1.4 (0.7)	<.001
Median (IQR)	1.0 (1.0–2.0)	2.0 (1.0–2.0)	2.0 (1.0–2.0)	
Very good or excellent review rate, mean (SD), %^c^	80.2 (35.2)	85.4 (29.7)	83.5 (31.9)	<.001

a *P* values are from *t* tests (for means) or χ^2^ tests (for categories).

b Turnaround times were not available for 52 manuscripts in the prepandemic period and 64 in the pandemic period.

c Reviewer ratings were not available for 55 manuscripts in the prepandemic period and 65 in the pandemic period.

**Table 2.** Characteristics of Manuscripts That Did or Did Not Address COVID-19

Characteristic	Not COVID-19 related (n = 2238)	COVID-19 related (n = 915)	Total (N = 3153)	*P* value^a^
Manuscripts reviewed per week, No.				
Mean (SD)	31.9 (10.0)	13.1 (5.8)	46.4 (12.2)	<.001
Median (IQR)	31 (26–37)	13 (8–18)	45 (37–53)	
Type of submission, No. (%)				
Transfer	1368 (61.1)	584 (63.8)	1952 (61.9)	.16
Direct submission	870 (38.9)	331 (36.2)	1201 (38.1)	
Type of article, No. (%)				
Original investigation	1971 (88.1)	635 (69.4)	2606 (82.7)	<.001
Research letter	267 (11.9)	280 (30.6)	547 (17.3)	
Study design, No. (%)				
Clinical trial	185 (8.3)	41 (4.5)	226 (7.2)	<.001
Cohort	924 (41.3)	332 (36.3)	1256 (39.8)	
Cross-sectional	484 (21.6)	234 (25.6)	718 (22.8)	
Other design	645 (28.8)	308 (33.7)	953 (30.2)	
Reviewers invited, No.				
Mean (SD)	7.2 (4.6)	6.4 (4.4)	7.0 (4.6)	<.001
Median (IQR)	6.0 (4.0–10.0)	5.0 (3.0–8.0)	6.0 (4.0–9.0)	
Reviewers who accepted, No.				
Mean (SD)	1.7 (0.5)	1.8 (0.5)	1.8 (0.5)	<.001
Median (IQR)	2.0 (1.0–2.0)	2.0 (2.0–2.0)	2.0 (1.0–2.0)	
Invitation acceptance rate, %				
Mean (SD)	36.1 (27.2)	44.1 (30.1)	38.4 (28.3)	<.001
Median (IQR)	28.6 (16.7–50.0)	33.3 (20.0–66.7)	28.6 (16.7–50.0)	
Reviewers who declined, No.				
Mean (SD)	3.1 (3.0)	2.7 (2.8)	3.0 (2.9)	<.001
Median (IQR)	2.0 (1.0–5.0)	2.0 (1.0–4.0)	2.0 (1.0–4.0)	
Invitation decline rate, %				
Mean (SD)	35.2 (22.3)	32.8 (22.6)	34.5 (22.4)	.006
Median (IQR)	37.5 (20.0–50.0)	33.3 (14.3–50.0)	37.5 (20.0–50.0)	
Reviews returned, No.				
Mean (SD)	1.7 (0.6)	1.8 (0.5)	1.7 (0.5)	<.001
Median (IQR)	2.0 (1.0–2.0)	2.0 (2.0–2.0)	2.0 (1.0–2.0)	
Mean review turnaround time, d^b^				
Mean (SD)	14.6 (7.0)	13.7 (6.8)	14.4 (7.0)	.002
Median (IQR)	14.5 (10.0–18.5)	14.0 (9.0–17.5)	14.0 (10.0–18.0)	
Very good or excellent reviews, No.				
Mean (SD)	1.4 (0.7)	1.5 (0.7)	1.5 (0.7)	<.001
Median (IQR)	2.0 (1.0–2.0)	2.0 (1.0–2.0)	2.0 (1.0–2.0)	
Very good or excellent reviews, mean (SD), %^c^	85.1 (29.7)	85.9 (29.7)	85.4 (29.7)	.49

a *P* values are from *t* tests (for means) or χ^2^ tests (for categories).

b Turnaround times were not available for 54 non–COVID-19–related manuscripts and 10 COVID-19–related manuscripts.

c Reviewer ratings were not available for 55 non–COVID-19–related manuscripts and 10 COVID-19–related manuscripts.

## Data Availability

See the Supplement.

## Key Points

### Question

How did peer review change from before to during the first year of the COVID-19 pandemic at a large open-access general medical journal?

### Findings

In this cohort study of 5013 manuscripts reviewed and peer reviews invited by an open-access general medical journal, peer review turnaround time was slightly shorter and editor-reported review quality was modestly increased during the first year of the pandemic.

### Meaning

This study found that peer review at a large, open-access general medical journal remained resilient during the first 15 months of the pandemic despite shifts in in the dynamics of the review process.

## References

[1] Malicki M, Jeroncic A, Ter Riet G, Preprint servers’ policies, submission requirements, and transparency in reporting and research integrity recommendations. JAMA. 2020;324(18):1901–1903. doi:10.1001/jama.2020.17195 33170231 PMC7656281

[2] Paglione LD, Lawrence RN. Data exchange standards to support and acknowledge peer-review activity. Learn Publ. 2015;28(4):309–316. doi:10.1087/20150411

[3] Fox CW, Albert AYK, Vines TH. Recruitment of reviewers is becoming harder at some journals: a test of the influence of reviewer fatigue at six journals in ecology and evolution. Res Integr Peer Rev. 2017;2(1):3. doi:10.1186/s41073-017-0027-x 29451533 PMC5803623

[4] Rennie D, Flanagin A. Research on peer review and biomedical publication: furthering the quest to improve the quality of reporting. JAMA. 2014;311(10):1019–1020. doi:10.1001/jama.2014.1362 24618962

[5] Rennie D Let’s make peer review scientific. Nature. 2016;535(7610):31–33. doi:10.1038/535031a 27383970

[6] Helliwell JA, Bolton WS, Burke JR, Tiernan JP, Jayne DG, Chapman SJ. Global academic response to COVID-19: Cross-sectional study. Learn Publ. 2020;33(4):385–393. doi:10.1002/leap.1317 32836910 PMC7362145

[7] Squazzoni F, Bravo G, Grimaldo F, García-Costa D, Farjam M, Mehmani B. Gender gap in journal submissions and peer review during the first wave of the COVID-19 pandemic: a study on 2329 Elsevier journals. PLoS One. 2021;16(10):e0257919. doi:10.1371/journal.pone.0257919 34669713 PMC8528305

[8] Berkwits M, Flanagin A, Bauchner H, Fontanarosa PB. The COVID-19 pandemic and the JAMA Network. JAMA. 2020;324(12):1159–1160. doi:10.1001/jama.2020.18298 32960223

[9] Carroll AE. Peer review: the worst way to judge research, except for all the others. New York Times. Published November 5, 2018. Accessed October 25, 2021. https://www.nytimes.com/2018/11/05/upshot/peer-review-theworst-way-to-judge-research-except-for-all-the-others.html

[10] 2021 Release of Journal Citation Reports: Journals Ranked by Impact. Clarivate Analytics; 2021.

[11] von Elm E, Altman DG, Egger M, Pocock SJ, Gøtzsche PC, Vandenbroucke JP; STROBE Initiative. The Strengthening the Reporting of Observational Studies in Epidemiology (STROBE) statement: guidelines for reporting observational studies. Ann Intern Med. 2007;147(8):573–577. doi:10.7326/0003-4819-147-8200710160-00010 17938396

[12] JAMA Network instructions for authors. Updated December 14, 2022. Accessed December 29, 2022. https://jamanetwork.com/journals/jamanetworkopen/pages/instructions-for-authors

[13] WHO Director-General’s opening remarks at the media briefing on COVID-19—11 March 2020. Accessed January 8, 2022. https://www.who.int/director-general/speeches/detail/who-director-general-s-opening-remarks-at-the-media-briefing-on-covid-19---11-march-2020

[14] R Core Team. R: A Language and Environment for Statistical Computing [computer program]. R Foundation for Statistical Computing; 2019. Accessed January 8, 2022. http://www.R-project.org

[15] Dong E, Du H, Gardner L. An interactive web-based dashboard to track COVID-19 in real time. Lancet Infect Dis. 2020;20(5):533–534. doi:10.1016/S1473-3099(20)30120-1 32087114 PMC7159018

[16] Myers KR, Tham WY, Yin Y, Unequal effects of the COVID-19 pandemic on scientists. Nat Hum Behav. 2020;4(9):880–883. doi:10.1038/s41562-020-0921-y 32669671

[17] Kibbe MR. Consequences of the COVID-19 pandemic on manuscript submissions by women. JAMA Surg. 2020;155(9):803–804. doi:10.1001/jamasurg.2020.3917 32749449

