# Supplementary material for: Peer Review in a General Medical Research Journal Before and During the COVID-19 Pandemic
Source: JAMA Netw Open. Author manuscript; Available in PMC 2024 Feb 8. (PMC10851144; doi:10.1001/jamanetworkopen.2022.53296)
Supplement: Supplement [file NIHMS1958936-supplement-Supplement.pdf]

Data Sharing Statement

Perlis. Peer Review in a General Medical Research Journal Before and During the COVID-19 Pandemic. *JAMA Netw Open*. Published January 27, 2023.
doi:10.1001/jamanetworkopen.2022.53296

Data
Data available: No
